# Rate of the Reaction NO+N, and Some Heterogeneous Reactions Observed in the Ion Source of a Mass Spectrometer*

**DOI:** 10.6028/jres.065A.042

**Published:** 1961-10-01

**Authors:** John T. Herron

## Abstract

The rate of the reaction NO + N→ N_2_+O has been measured to be 1.0±0.5×10^13^ cm^3^ moles^−1^ sec^−1^ at room temperature. The heterogeneous reactions N+O→NO and O + O→O_2_ were observed to occur in the ion source of the mass spectrometer.

## 1. Introduction

The reaction of active nitrogen with nitric oxide has been studied mass spectrometrically by Kistiakowsky and Volpi [1,2].^1^ They concluded that the reaction taking place was

$$\text{NO}+N\to N_{2}+O$$ (1)

and that the rate constant for the reaction had a lower limit of about 5×10^13^ cm^3^ mole^−1^ sec^−1^ at room temperature.

In view of the importance of this reaction in certain atmospheric phenomena as well as its practical value as a method of measuring nitrogen atom concentrations, it has been re-examined using a mass spectrometer of high sensitivity.

In addition, a mass spectrometric study of active nitrogen has been made.

In one of the previous mass spectrometric studies, there was some evidence to suggest the presence of a highly energetic species other than nitrogen atoms [3]. This was not observed by a second group of investigators [4]. The present study was undertaken in the hope of resolving this difference.

## 2. Experimental Procedure

The mass spectrometer was a 60° sector type instrument of 12-in. radius of curvature. Magnetic scanning was used at a constant ion accelerating potential of 5,000 v.

The gas inlet system and ion source were so designed that gas could pass directly from the reactor or discharge zone into the electron beam of the mass spectrometer. Thus a certain fraction of the gas entering the ion source could be ionized without suffering wall collisions in the ion source.

Ion currents were measured by means of a 14 stage ion multiplier, vibrating reed electrometer, and pen recorder. The minimum detectable signal was about 10^−18^ amp.

Active nitrogen was produced by passing dry nitrogen through a 2450 Mc/s electrodeless discharge. Two types of flow system were used, one for studying active nitrogen, and another for studying its reactions. In the former, gas at about 0.1 mm total pressure was taken directly from the discharge zone, through a 1 mm diameter “leak” leading to the mass spectrometer ion source which was about 10 cm from the leak. The appearance potentials of the ions produced in the ion source by electron impact were measured by varying the electron energy in 0.2 ev steps.

**Figure 1:**
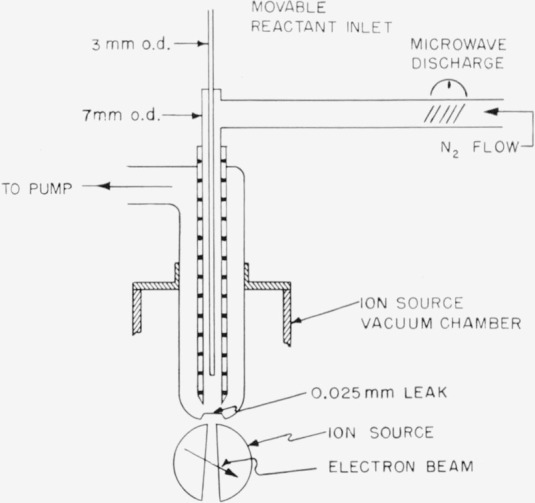
Reactor.

The reaction of active nitrogen with nitric oxide was studied in the reactor shown in figure 1. Nitric oxide entered the reactor through the movable central tube, and active nitrogen through the outer tube. Gas leaving the reactor passed over a 0.025 mm diameter leak for continuous sampling by the mass spectrometer.

Although the reactor was equipped with a heater, all the experiments reported here were made at room temperature.

In any given experiment, all experimental conditions were fixed except the initial nitric oxide concentration. The range of experimental conditions used in the various experiments was, pressure, 0.33 to 0.87 mm, nitrogen flow, 16 to 65 cm^3^/min, length of reaction zone, 1.1 to 2.7 cm, and reaction time, 0.5×10^−3^ to 1.7×10^−3^ sec.

Partial pressures of stable reactants and products were measured by conventional mass spectrometric techniques using an ionizing energy of 50 ev. Relative nitrogen atom partial pressures were measured using a nominal ionizing energy of 24 ev, i.e., at sufficiently low energy to minimize formation of N^+^ ions from dissociative ionization of nitrogen. Small corrections were made for contributions of N^+^ ions from nitric oxide. The relative values were related to absolute units of pressure by equating the N^+^ signal due to nitrogen atoms (with no reactant present) to the limiting amount of nitric oxide consumed via reaction (1), assuming this to be the correct stoichiometry.

In some experiments 96 percent N^15^O was used as a reactant instead of normal N^14^O.

## 3. Results and Discussion

**Figure 2:**
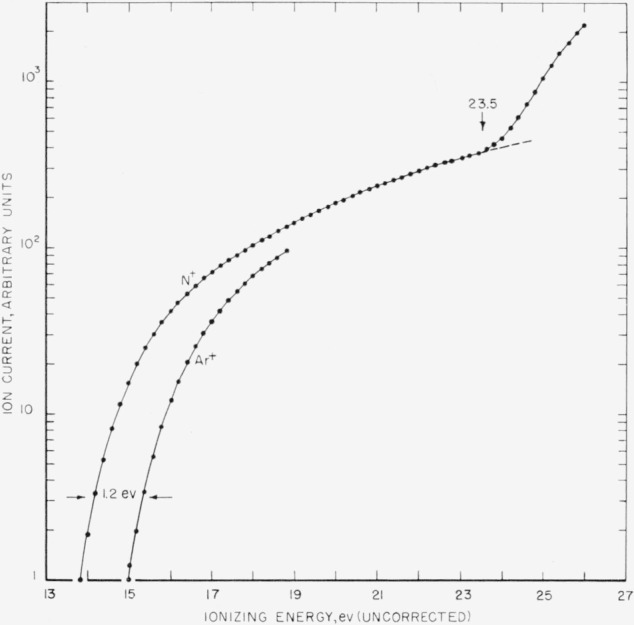
Ionization efficiency curve for the *N*^+^ ion from active nitrogen.

The ionization efficiency curve for the N^+^ ion produced by electron impact from active nitrogen is shown in figure 2. The ionization potential of argon, 15.76 ev [5], was used to calibrate the energy scale.

The lowest N^+^ appearance potential was 14.53±0.12 ev. This is equal to the ionization potential of the ground state nitrogen atom, 14.54 ev [5]. The sharp break upwards in the curve at 23.5 ev corresponds to the formation of N^+^ ions from N_2_. The break in the ionization efficiency curve at 16.1 ev, reported by Jackson and Schiff [3] was not observed. However, the N_2_^+^ ionization efficiency curve did show a small shift (less than 1 ev) to lower energy when the discharge was turned on. This may have been due to vibrationally excited nitrogen molecules which have been reported to be present in active nitrogen [6, 7].

It seems safe to conclude that the only reactive species involved in the nitric oxide reaction under the conditions of the present work are ground state nitrogen atoms.

Since the nitric oxide is in its ground state at room temperature, the reaction under study can be more properly written as

$$\text{NO}({\;}^{2}\text{II})+N({\;}^{4}S)\to N_{2}+O.$$

The energetic states of the products are not known, although there is some evidence to suggest that the N_2_ is in a vibrationally excited state [6]. This is not surprising, since the reaction is 80 kcal exothermic.

**Figure 3:**
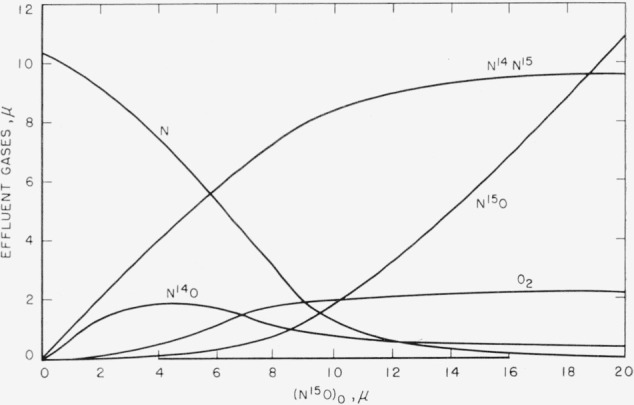
Steady state partial pressures of effluent gases as a function of initial *N*^15^*O* partial pressure.

Although experiments were made with both N^14^O and N^15^O, it is more illustrative to discuss the results in terms of the N^15^O experiments. A typical set of results is shown in figure 3.

The products of the reaction are N^14^N^15^, N^14^O, and O_2_. Other than confirming its presence, no attempt has been made to analyze for atomic oxygen. Over the range of initial N^15^O partial pressures used, the partial pressure of N^14^N^15^ produced was equal to the amount of N^15^O consumed, as would be expected from eq (1). This was not exactly true for the change in partial pressure of nitrogen atoms, although the uncertainty in nitrogen atom measurements was considerably greater.

The second order rate equation for the reaction can be written as

$$\log\;\left\{\frac{N_{0}({\text{NO}}_{0}-x)}{{\text{NO}}_{0}(N_{0}-x)}\right\}=\frac{k}{2.303}({\text{NO}}_{0}-N_{0})t$$

where N_0_ and NO_0_ are the initial nitrogen atom and nitric oxide concentrations respectively, *x* is the extent of reaction, and *t* the reaction time.

**Figure 4:**
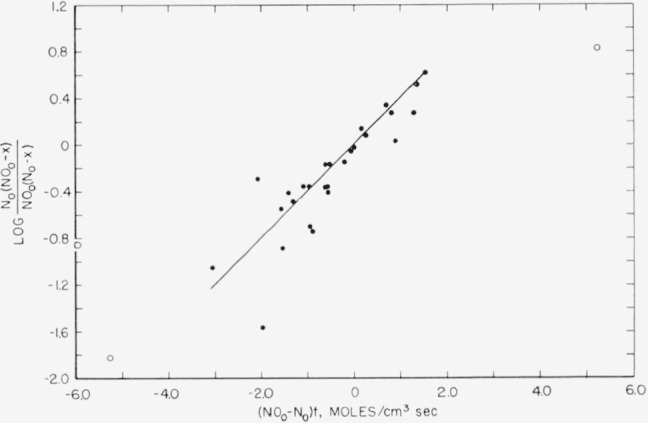
Summation of rate data for the reaction *NO*+*N*

A plot of log [N_0_(NO_0_−*x*)]/[NO_0_(N_0_−*x*)] versus (NO_0_−N_0_)*t* gives a straight line, the slope of which, as determined by a least squares fit of the data, yields *k*. The best value of *k*, taken as the un-weighted average of 6 experiments was *k*=(1.0±0.5)×10^13^ cm^3^ mole^−1^ sec^−1^. The deviation given is 3 times the standard deviation of the average. All the data for the 6 separate experiments are plotted together in figure 4. Points shown in open circles were not used in determining *k*.

The major source of uncertainty in this value is due to incomplete mixing of the reactants, since the time for a molecule to diffuse across the reactor is comparable with the reaction time.

Under conditions of constant flow of nitrogen and nitric oxide, it was found that the distance between nitric oxide inlet and leak had no effect on the nitric oxide partial pressure until the distance was 1 cm or less. The average distance used in the experiments was 2.1 cm.

The value of *k* given above agrees, within the limits of experimental error, with that calculated from the data of Clyne and Thrush for the reaction at 300 °K, i.e., *k*=(2.2±0.6)×10^13.0±0.5^ cm^3^ mole^−1^ sec^−1^.

The N^14^O and O_2_ observed as products of the reaction are probably due to secondary reactions. The rate controlling steps for homogeneous reactions giving rise to these products would involve termolecular reactions, which, under the conditions of this work, would be too slow to account for the amount of products observed.

It seems almost certain that these reactions are occurring on the walls of the mass spectrometer ion source. If this is the case, then the partial pressures of N^14^O and O_2_ shown in figure 3, have no absolute significance, but are relative partial pressures within the ion source. With a total pressure in the reactor of 0.5 mm the pressure at a point just below the ion source housing was about 10^−6^ mm. The pressure in the ion source probably would be no greater than 5×10^−6^ mm, so that the pressure differential between reactor and ion source is about 10^−5^. Therefore, the absolute partial pressures of N^14^O and O_2_ in the ion source were of the order of 10^−8^ mm. As might be expected at such low pressures, these heterogeneous reactions appear to follow second order kinetics.

It should be noted that atomic recombination in the ion source has been postulated previously to explain the low sensitivity of highly reactive atomic species such as N or O [1,2,9]. Under the usual experimental conditions, however, the recombination product is inseparable from the original molecules used as a source of atoms. Similarly, in the present work, N_2_ formed in the ion source was not separable from that passing unchanged through the discharge.

## Footnotes

1 Figures in brackets indicate the literature references at the end of this paper.
